# A Novel miR-451a isomiR, Associated with Amelanotypic Phenotype, Acts as a Tumor Suppressor in Melanoma by Retarding Cell Migration and Invasion

**DOI:** 10.1371/journal.pone.0107502

**Published:** 2014-09-19

**Authors:** Sankhiros Babapoor, Elizabeth Fleming, Rong Wu, Soheil S. Dadras

**Affiliations:** 1 Department of Genetics and Developmental Biology, University of Connecticut Health Center, Farmington, Connecticut, United States of America; 2 CICATS Biostatistics Center, University of Connecticut Health Center, Farmington, Connecticut, United States of America; 3 Department of Dermatology, University of Connecticut Health Center, Farmington, Connecticut, United States of America; University of Tennessee, United States of America

## Abstract

miRNAs are key regulatory small non-coding RNAs involved in critical steps of melanoma tumorigenesis; however, the relationship between sequence specific variations at the 5′ or 3′ termini (isomiR) of a miRNA and cancer phenotype remains unclear. Deep-sequencing and qRT-PCR showed reduced expression of miR-144/451a cluster and most abundant isomiR (miR451a.1) in dysplastic nevi, in-situ and invasive melanomas compared to common nevi and normal skin (n = 101). miRNA in situ hybridization reproducibly confirmed lost miR-451a.1 in melanoma compared to nevus cells or adjacent keratinocytes. Significantly higher expression of miR-451a.1 was associated with amelanotic phenotype in melanomas (n = 47). In contrast, miR-451a was associated with melanotic phenotype, absent pagetoid scatter of intraepidermal melanocytes, superficial spreading histological subtype and tumor inflammation. Sequencing miRNAs from cultured melanocytes with cytoplasmic melanin gradient (light, medium to dark) showed absent miR-451a while revealing other melanin-associated miRNAs, e.g. miR-30b, miR-100 and miR-590 in darkly and let-7a, let-7i and let-7f in lightly to moderately pigmented cultured melanocytes. Ectopic expression of miR-144/451a in melanoma cell lines resulted in markedly higher levels of mature miR-451a.1 than miR451a or miR-144; and significantly retarded cell migration and inhibited invasion in a glucose-sensitive manner. Surprisingly, these effects were not mediated by calcium binding protein 39 (CAB39), a proven miR451a gene target. miR-144/miR-451a cluster is a novel miRNA locus with tumor suppressive activity in melanoma.

## Introduction

The incidence and mortality of melanoma have continually increased over the past decades in the US. It is estimated that 76, 690 individuals were diagnosed with new cases and 9,480 were deaths (men and women) of cutaneous melanoma in 2013 [1]. Notwithstanding the significant discoveries in the genetic aspects of melanoma research in the last two decades, e.g. BRAF activating mutations [2] and other genetic alterations [3], the melanoma epigenome remains poorly understood (reviewed in [4]).

miRNAs are endogenous, ∼22 nucleotide non-coding small RNAs, which can regulate gene expression in animals and plants by complementary base-pairing to the mRNAs of target genes to specify mRNA cleavage or translation repression [5]. Evidence supporting the role of miRNAs in melanoma include: 1) disruptions of miRNA coding sequence (or binding site) via inherited variants or somatic mutations in the 3′ untranslated regions of *KIT* [6] and *KRAS* [7] oncogenes are strong genetic markers for melanoma risk; 2) deregulated expression of enzymes (e.g. Dicer) participating in canonical miRNA biogenesis pathway [8], globally altering the pool of mature miRNAs and 3) functional relevance of specific miRNAs deregulated in growth and invasion in cell models [9], [10], [11], [12] and in melanoma metastases [13].

Recently, we established a full repertoire of human miRNA transcriptome by deep-sequencing small non-coding RNAs (18–30 nt) directly in biopsies of melanocytic nevi (benign melanocytic hyperplasia), thick primary (>4.0 mm) and metastatic melanomas with matched normal skin in parallel to melanocytes and melanoma cell lines [14]. These results not only defined a set of top-40 miRNAs, which properly classified nevus from melanoma, but also demonstrated extensive sequence variations (isomiRs), revealing a higher complexity of different miRNA populations in melanoma that may participate in clinically relevant gene regulatory networks [14]. isomiRs [15] are miRNA sequences exhibiting variations in the 5′ and 3′ termini and some along the miRNA length from their described reference in miRBase, miRNA reference database [16]. It has been demonstrated that isomiRs are not artifacts generated from deep sequencing, rather mature variants that result in different cleavage sites for RNASEN and Dicer [17], enzymes in miRNA biogenesis pathway. isomiRs have been identified in a variety of cell types including peripheral blood monocytes [18], oocytes [19], neurons [20] and epidermal melanocytes and keratinocytes and melanoma cells [14]. isomiRs, found across animal species, are thought to result from evolutionary pressures where the dominant isoforms are expressed as advantageous sequences in response to functional/environmental pressures [21]. Thus to have an in-depth understanding of miRNA biology particularly in cancer, it is important to determine the relationship between dominant isoform sequences and specific cancer phenotype and/or clinical outcome.

While unclear how miRNAs might regulate cutaneous melanin pigment synthesis, melanogenesis is regulated by a complex set of hormonal, receptor-mediated and receptor independent factors. Melanin is synthesized by melanocytes within cellular organelles (melanosomes). Melanosome development occurs in four steps: early matrix organization (stage I), organized matrix without melanin (stage II), deposition of melanin (sage III) and fully melanized and completely filled with melanin (stage IV) (reviewed in [22]). Microphtalmia-associated transcription factor (MITF) plays a central role in regulation of the genes involved in melanin synthesis and transfer; and pigmentation plays an important as the crossroad between genetic and environmental factors contributing to melanoma (reviewed in [23]. For example, in cultured hamster melanoma cell lines, L-tyrosine induced melanin synthesis in a dose- and time-dependent manner, suggesting that L-tyrosine and L-DOPA, as consecutive substrates and intermediates of melanogensis, can autoregulate mammalian pigmentation (reviewed in [24]). Altering melanogenic activity by using melatonin (as tyrosinase inhibitor) has been proposed as a treatment strategy for melanoma [25]. Moreover, MITF is a target of the MAPK pathway [26] and has recently been shown to mediate resistance to RAF-MEK-ERK inhibitor therapy [27].

We identified that the most abundant isomiR sequences for 6 out of 10 top miRNAs deregulated in cutaneous melanoma—miR-205, miR-211, miR-15b, miR-26a, miR-203, let-7i, miR-142, miR-150, miR-146a and miR-451a—did not match as abundant sequences in miRBase (v18) [14]. Among these, miR-451a was found to be in a longer pre-miRNA as miR-144/451a cluster required for erythroid homeostasis [28] and miR-451a expressed during human erythropoiesis [29]. Although involved in normal hematopoiesis, miR-451a functions as a tumor suppressor in several human malignancies including gastrointestinal cancer cells [30], glioma cells [31] and non-small cell lung cancer [32]. In glioma cells, miR451a regulates liver kinase B1 (LKB1)/5′ adenosine monophosphate-activated protein (AMPK) signaling pathway by targeting CAB39 (MO25α) responding to cellular metabolic stress [31] and indirectly inhibiting phosphatidylinositol 3-kinase (PI3K)/AKT pathway [33]. In this study, we demonstrated a tumor suppressive role for a novel isomiR of miR-451a (miR-451a.1) in melanoma progression and histopathological association with amelanotic phenotype.

## Results

### Down-regulation of miR-144/451a in melanoma

We applied next-generation sequencing (NGS) platform to carry out an in-depth analysis of miRNA transcriptome in biopsies of common nevi (CN) and primary cutaneous melanomas (PCM) and defined a set of top-40 list, which properly classified normal from cancer [14]. Gene Expression Omnibus (GEO) has accepted the deep-sequence results and dataset (GSE36236). In this analysis, miR-144/451a cluster ranked among the top-40 miRNAs and showed a 3-fold reduction (% per total miRNA) of miR451a and miR-144-3p in PCM compared to NS; and absent from melanocytes and melanoma cell lines (Table S1 in File S1). Comparing to miR-144-3p, miR451a was more abundantly present in all samples and constituted 2.1% of total miRNAs in NS.

**Figure 1:**
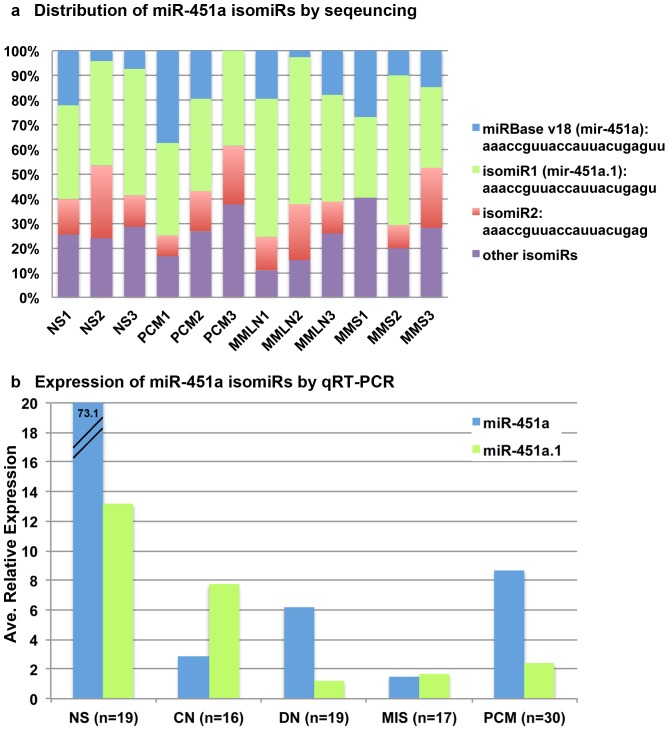
Distribution and expression of miR-451a isomiRs by NGS and qRT-PCR. (a) miR-451a.1 (isomiR1) was the most abundant sequence among normal skin (NS), primary cutaneous melanoma (PCM), metastatic melanoma to LN (MMLN) and to skin (MMS). (b) In a validation cohort, qRT-PCR showed significantly reduced levels for both miR-451a and miR-451a.1 in PCM compared to NS. miR-451a.1 levels were decreased in DN, MIS and PCM compared to CN. The relative expression was an average RQ values for all samples. qRT-PCR were performed in triplicates for every sample.

miRDeep 2.0 analysis mapped the sequence reads to the UCSC reference genome GRCh37 (browser hg19), compiling stretches of a miR-451a precursor sequences that demonstrated sequence variations in the 5′ and 3′ termini and in some cases nucleotide substitutions along the miRNA length (isomiRs). Surprisingly, the miR-451a sequence for which commercial qRT-PCR assays are available did not match the most abundant sequence in miRBase (v18 or v17) (reads of 8 and 53, respectively). In fact, the most abundant sequences were isomiR1 and isomiR2 (reads of 534 and 371, respectively), exemplified in NS3 library with total of 1260 counts (Figure S1 in File S1); a similar pattern was also noted in PCM5 library with total of 149 read counts (Figure S2 in File S1), showing a 8.5-fold reduction in total counts for all miR-451a isomiRs. To confirm these sequence variations, we compared the distribution of normalized copy number of miR-451a isomiRs in the libraries of NS, PCM, MMLN and MMS. This comparison showed that isomiR1 (which we named miR-451a.1) was the most abundant sequence and not miRBase v18 (miR-451a) (Figure 1a). We custom ordered primer sets based on miR-451a and miR-451a.1 sequence (Table S2 in File S1) to specifically validate the expression of these isomiRs by quantitative real time-PCR (qRT-PCR) in an independent series of NS (n = 19), CN (n = 16), dysplastic nevi (DN, n = 19), melanoma in situ (MIS, n = 17) and PCM (n = 30) (Table S3 in File S1). The qRT-PCR results showed a significant reduced expression for both miR-451a (8.6, *P* = 0.0001) and miR-451a.1 (2.3, *P* = 0.0006) in PCM compared to NS (73.1 and 13.1), respectively (Figure 1b). Compared to CN, expression of miR-451a was modestly increased in DN and PCM, but not MIS whereas the expression of miR-451a.1 was progressively decreased in DN, MIS and PCM (Figure 1b), not reaching a statistical significance (Table S4 in File S1).

**Figure 2:**
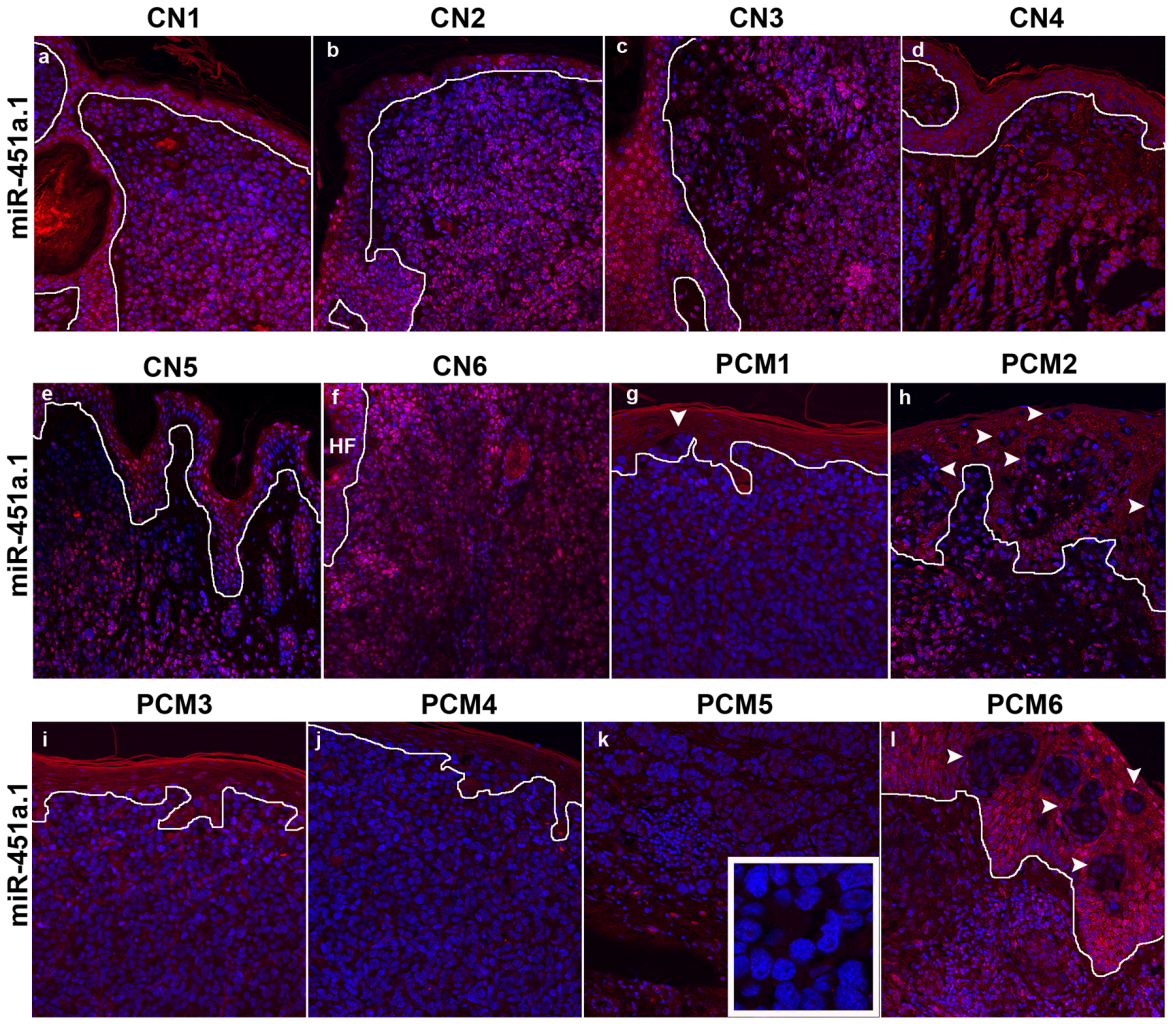
Decreased expression of miR-451a.1 in primary melanoma by MISH. (a-f) miR-451a.1 signal (red) was consistently detected in nuclei and cytoplasm of dermal nevus cells, overlying epidermal keratinocytes and epithelium of hair follicle (HF) in 6 representative CN; (g-i) this signal was absent from the nuclei and cytoplasm of dermal melanoma cells in 6 representative PCM. Loss of miR-451a.1 expression was best seen in the melanoma in situ cells (arrow heads) in contrast to surrounding keratinocytes. The solid line represents epidermal-dermal junction. All images were acquired under constant parameters. The original magnification was 200× for all images; inset was 630×.

To confirm down-regulation, we examined miR-451a.1 expression by miRNA in situ hybridization (MISH) in another independent series of PCM and CN (n = 23) and detected a reproducible nuclear and cytoplasmic miR-451a.1 expression in the nevus cells and the overlying epidermal keratinocytes (Figure 2a-f), absent in melanoma cells and the overlying keratinocytes (Figure 2g-i) in a series of 6 representative CN and PCM biopsies. miR-451a.1 was consistently expressed in the nuclei and some in the cytoplasm of epidermal keratinocytes in NS while it was absent in invasive (dermal) melanoma cells (Figure S3a-c in File S1) and in scramble control (Figure S3d-g in File S1). We detected a robust U6 (small RNA positive control) nuclear signal in the dermal nevus cells and the overlying epidermal keratinocytes, which was absent in scramble control (Figure S3h-I in File S1); no signal was detected in scramble control CN or PCM sections (Figure S4a-f in File S1). These results showed that the expression of miR-451a.1, an abundant isomiR in normal skin, progressively decreased in melanoma (in situ and invasive) and dysplastic nevi compared to common nevi.

### Higher miR-451a.1 expression and amelanotic phenotype

**Figure 3:**
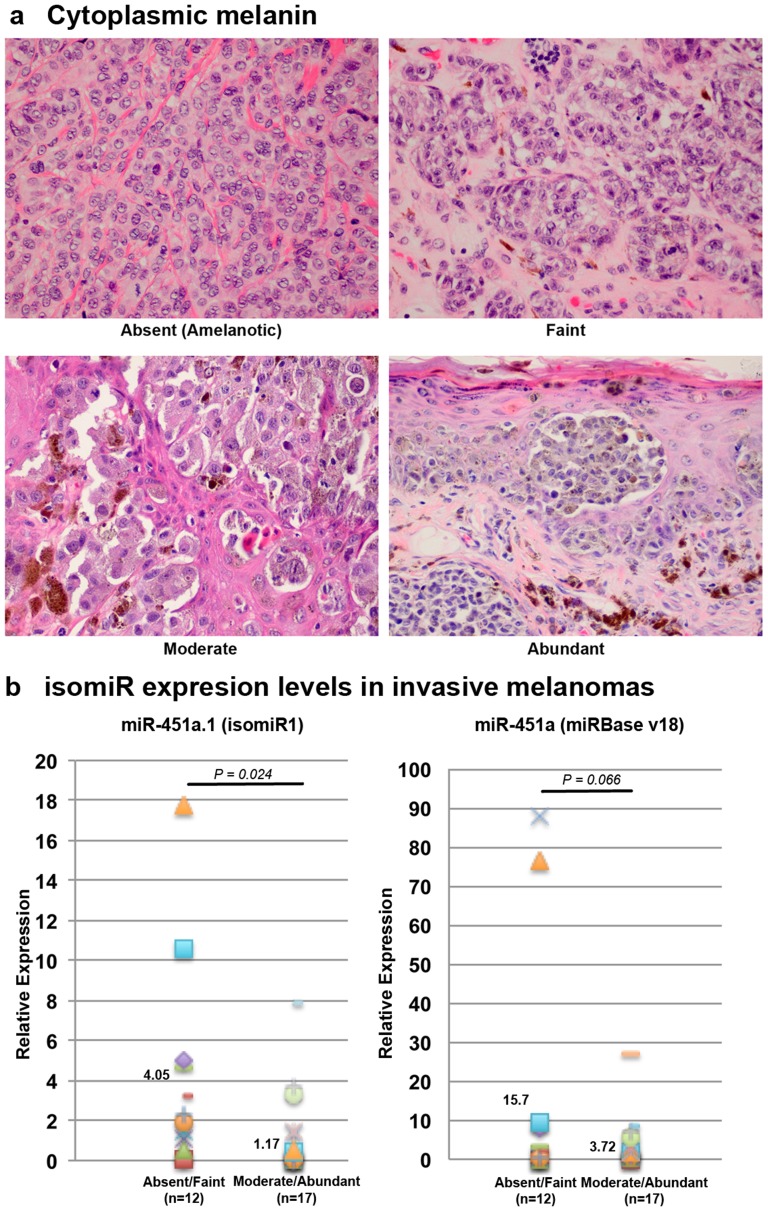
Higher miR-451a.1 expression was associated with amelanotic phenotype in melanoma. (a) Based on melanoma intracytoplasmic melanin, PCM biopsies were classified into absent, faint, moderate or abundant. (b) The expression of miR-451a.1 was significantly higher in amelanotic lesions, in contrast to miR-451a (miRBase v18). The numbers shown in the dot-plots are the average RQ values.

Given the abundance of mature miR-451a.1 over miR-451a, we asked whether this isomeric difference was associated with melanoma histopathologic characteristics (Table S3 in File S1). We compared the expression of miR-211, miR-451a.1 and miR-451a with histopathological characteristics in PCM (n = 30) and MIS (n = 17) (Table S5 in File S1). Higher expression of miR-451a.1 (*P* = 0.024) was significantly associated with absent to faintly pigmented PCM (Figure 3) whereas both miR-451a and miR-451a.1 were associated with less melanin in MIS (Table S5 in File S1). miR-451a was associated with superficial spreading melanoma subtype, tumor inflammation and absence of upward scatter of intraepidermal melanocytes (Table S6 in File S1). The expression of miR-451a.1, not miR-451a, was reduced ∼4-fold in DN compared to CN. Comparing the expression of miR-451a.1 and miR-451a to cytological atypia (slight vs. moderate/severe) in DN (n = 19) did not reach statistical significance (*P* = 0.1496 and 0.8932, respectively).

**Figure 4:**
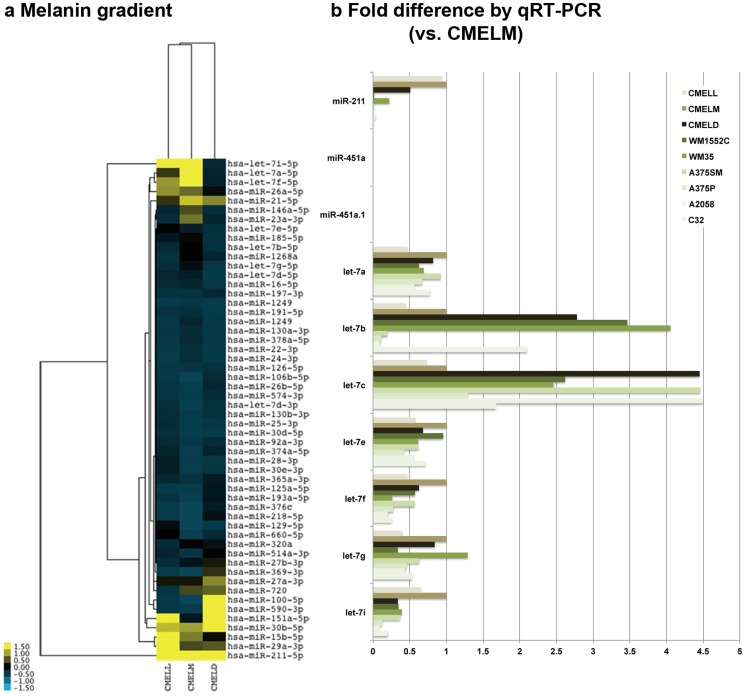
Expression of melanin-related miRNAs. (a) Unsupervised clustering heat map of NGS results demonstrated a relationship between melanin gradient and miRNA expression in cultured melanocytes that were lightly (CMELL), moderately (CMELM) and darkly (CMELD) pigmented. (b) qRT-PCR results showed differential expression of the let-7 family according to melanin, compared to melanoma cell lines: WM1552C, WM35, A375SM, A375P, A2058 and C32.

Given the inverse correlation between melanin pigmentation and miR-451a.1, we compared miRNA NGS results from three primary cultured melanocytes —isolated from three individuals with light (CMELL), medium (CMELM) and dark (CMELD) skin—according to their melanin content. A clustering analysis showed higher expression of let-7i, let-7a and let-7f in CMELM and CMELL than CMELD; and higher expression of miR-100, miR-590, miR-30b in CMELD than in CMELL and CMELM (Figure 4a). qRT-PCR confirmed the melanin-associated expression of let-7i, let-7a and let-7f and revealed a differential expression pattern of let-7b, let-7c according to melanin (Figure 4b). miR-211 expression was dramatically reduced in melanoma cell lines compared to melanocytes; and both miR-451a and miR-451a.1 were absent in all tested cells. Overall, miR-451a.1 was associated with amelanotic and miR-451a with melanotic phenotypes in melanoma; and specific members of let-7 family with melanin gradient in cultured melanocytes.

### Biological role of miR-451a in tumor progression

**Figure 5:**
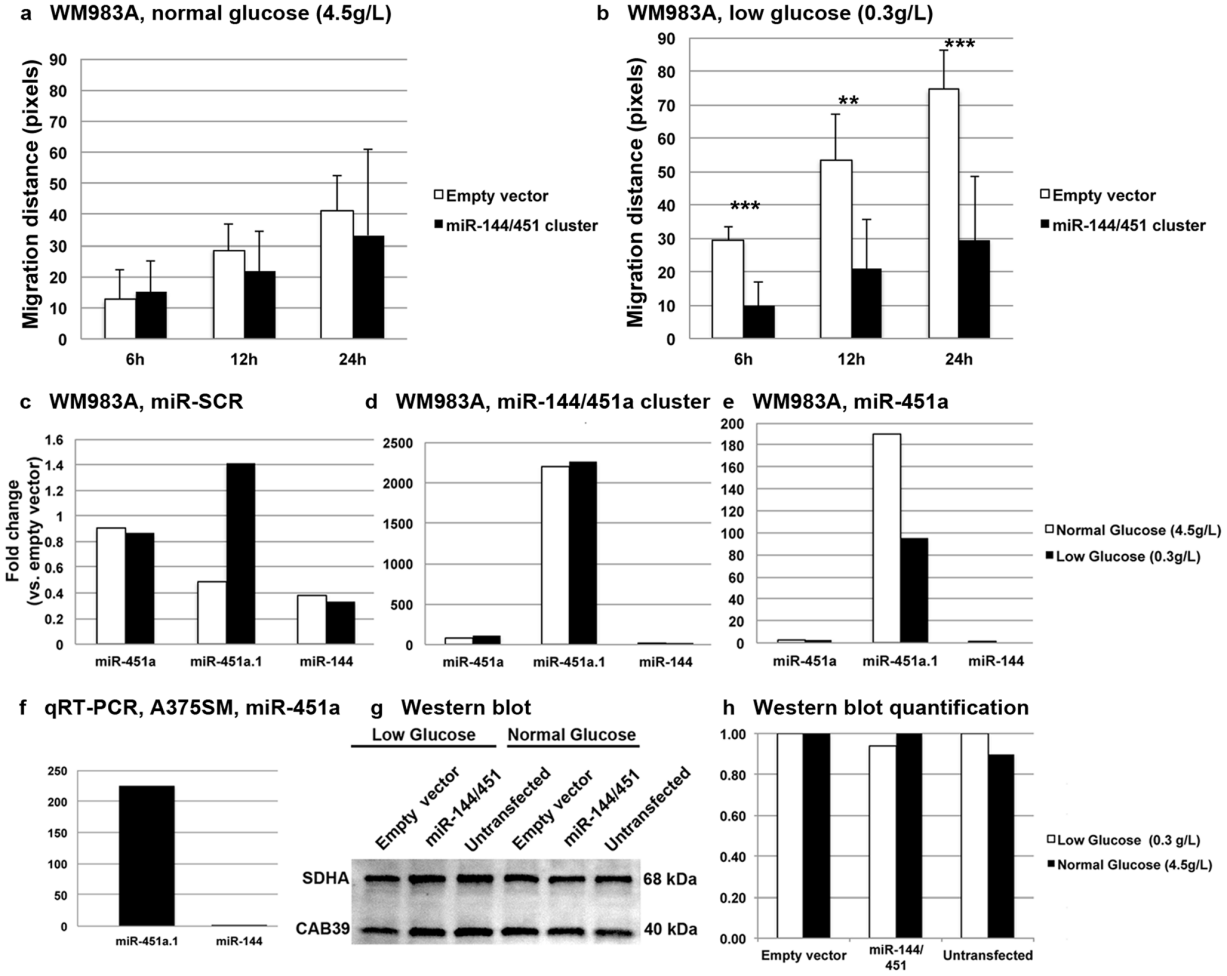
Expression of miR-144/451a cluster retarded migration of melanoma cells in a glucose sensitive manner. (a and b) Expressing miR-144/451a significantly decreased migration distance after 6, 12 and 24 h in a glucose-sensitive manner. (c) miR-451a.1 was the most abundant isomiR in scramble transfected (miR-SCR) control cells. (d) Expressing miR-144/451a led to>2000-fold increase in miR-451a.1 whereas (e) expressing miR-451a alone led to>180-fold in miR-451a.1. (f) Similarly, expressing miR451a alone led to>200-fold miR-451a.1 in another cell line (A375SM). (g and h) miR-144/451a did not alter the CAB39 protein levels. The error bars represent standard deviation from at least three different independent experiments. **P-value<0.005; ***P-value<0.0005. qRT-PCR were performed in triplicates.

We ectopically expressed miR451a using three different expression vectors: miR-144/451a cluster (containing both miR-451a and miR-144), miR-451a or miR-144 in three cell lines: WM983A (non-aggressive primary), A375SM and A375P (aggressive metastatic). Expressing miR-144/451a significantly decreased the migration distance of WM983A cells after 6, 12 and 24h in low glucose medium (0.3 g/L) compared to control (Figure 5a-b). To isolate the effect of miR-451a from miR-144, we transfected WM983A cells with miRNA scramble control (miR-SCR), miR-144/451a and miR-451a, then measured the expression of mature miR-144, miR-451a and miR-451a.1 by qRT-PCR (Figure 5c-e). These results showed expressing miR-144/451a led to>2000-fold increase in miR-451a.1 levels compared to miR-451a or miR-144 independent of glucose concentrations whereas miR-451a alone led to>180-fold in miR-451a.1 levels in normal glucose and ∼100-fold in low glucose medium (Figure 5d-e), showing that the cluster led to a more efficient processing and a significantly higher miR-451a.1 levels. In fact, the gene map for this cluster showed a longer pre-miRNA miR-144/451a (>300 bp) than either miR-451a or miR-144 alone (not shown). Similarly, expressing miR451a alone in another cell line (A375SM) led to>200-fold in miR-451a.1 (Figure 5f), confirming that miR-451a.1 is the most dominant isomiR after transfection and processing of pre-miRNA miR-144/451a. Surprisingly, expressing miR-144/451a did not alter CAB39 protein levels, a known gene target for miR-451a, in WM983A cells in either low or normal glucose medium (Figure 5g-h).

**Figure 6:**
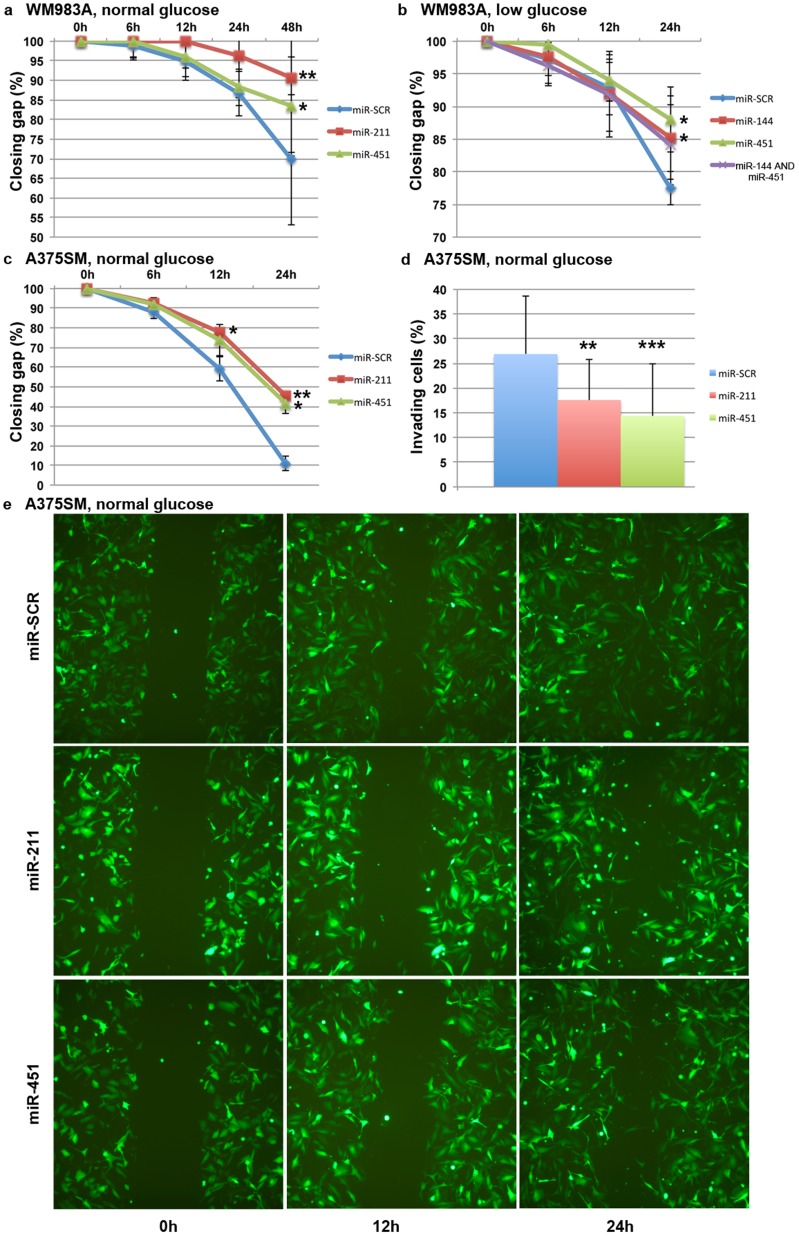
Expressing either miR-211 or miR-451a retarded migration and invasion of melanoma cells. (a) miR-211 or miR-451a significantly reduced the migration rate of WM983A cells after 48 h in normal glucose (4.5 g/L). (b) Lowering glucose (0.3 g/L) dramatically retarded migration rate of miR-451a or miR-144-transfected WM983A cells after 24 h; expressing both miR-144 and miR-451a together did not result in an added effect of retarded migration. (c) Moreover, expressing either miR-211 or miR-451a reduced the migration rate and (d) invasion of A375SM cells, respectively. (e) Cells transfected with miR-SCR nearly closed the gap by cross migration after 24 h whereas this process was retarded by miR-211 and miR-451a. The error bars represent standard deviation from at least two different independent experiments. *P-value<0.05; **P-value<0.005; ***P-value<0.0005.

We measured the effect of miR-451a or miR-211 expression in retarding migration rates in WM983A cells compared to control (miR-SCR) after 48h in normal glucose. Even though both mir-451a and miR-211 significantly reduced the migration rates of melanoma cells, miR-211 was more efficient (Figure 6a). Comparing the rates of cell migration between miR-144, miR-451a or miR-144 and miR-451a combined showed significantly retarded migration by expressing either miR-144 or miR-451a in low glucose concentration after 24 h; however, expressing both did not produce an additive effect (Figure 6b). Using another cell line (A375SM), expressing miR-451a or miR-211 significantly retarded the migration rate of melanoma cells (Figure 6c). In addition, we tested whether other discovered top-40 melanoma miRNAs, e.g. miR-203 or miR-205, could retard cell migration compared to miR-211 in two independent experiments using A375SM cell line. The results consistently showed that expressing miR-205 (test 1, *P* = 0.002 and test 2, *P* = 0.0005) or miR-211 (test 1, *P* = 0.019 and test 2, *P* = 7.13×10^−5^) significantly reduced the migration rate of A375SM cells compared to control after 24 hours of transfection whereas expressing miR-203 resulted in marked cell death (results not shown). Expressing miR-451a significantly reduced invasion in A375SM cells, as did miR-211 (Figure 6d); similarly, WM983A cell line transfected with miR-144/451a cluster showed 50% reduction in cell invasion (results not shown). For all the performed migration assays, we took representative images of at least 6 fields per time point in green cells to ensure that gap closure for only those cells expressing green fluorescent protein (GFP) and miRNA of interest are measured. For example, these results clearly demonstrated a retarded, time-dependent gap closure in cells expressing either miR-451a or miR-211 compared to control after 24 h (Figure 6e). Thus, expressing miR-144/miR-451a led to a more efficient processing and much higher levels of miR-451a.1, suggesting that this abundant isomiR, not miR-451a, inhibited melanoma cell migration and invasion, not mediated by CAB39.

## Discussion

NGS has demonstrated a higher level of complexity of miRNA transcriptome where different miRNA populations reveal isomiRs. Although isomiRs are found in a variety of cell types, the biological relationship between dominant sequence and specific cancer phenotype has not been investigated. Studying this isomeric relationship is a significant step towards a more in-depth understanding of the role of miRNAs in cancer. To this end, we investigated the role of a novel miRNA locus, miR-144/miR-451a cluster and its most abundant isomiR (miR-451a.1) in melanoma progression. NGS-based profiling of small RNAs revealed extensive isomeric differences in read counts of 6 out of top-10 deregulated miRNAs in melanoma specimens. The most abundant of isomiRs for miR-205, miR-211, miR-15b, miR-26a, miR-203, let-7i, miR-142, miR-150, miR-146a and miR-451a were not represented as the dominant seqeunces in miRBase (v18) [14]. Given that most microarrays and commercially available qRT-PCR assays rely on miRBase sequence data, lacking isomeric information could negatively impact miRNA discovery.

This notion was supported by the clinical findings that higher expression of miR-451a.1 was significantly associated with amelanotic phenotype whereas miR-451a was associated with melanotic phenotype, absent pagetoid scatter of intraepidermal melanocytes, superficial spreading histological subtype and tumor inflammation. The relationship between miR-451a (but not its isomiR, mi-R451a.1) and melanogenesis may provide a novel molecular basis for the observed relationship between strongly pigmented melanoma phenotype and patient overall and disease-free survival [34].

Moreover, our results also showed that miR-451a.1, compared to miR-451a, played a significant role in melanoma progression *in vitro*. Given that miR-144/451a are co-expressed from a common transcript [35], we posit that the significantly higher levels of mature miR-451a.1, compared to miR-451a and miR-144, after transfection by miR-144/451a cluster vs. miR-451a or miR-144 alone, are due to a more efficient processing which resulted in a higher abundance of miR-451a.1. Thus significantly higher (>2000-fold increase) levels in miR-451a.1 compared to miR-451a or miR-144 is most likely responsible for retarded cell migration and inhibited cell invasion. To ensure that mir-144/451a transfection produced no off-target effects, we compared the rates of retarded cell migration to other miRNAs such as miR-203, miR-205 and miR-211. These results showed the efficiency of reducing cell migration in a decreasing order as miR-211> miR-451a.1> miR-205; and miR-203 resulted in marked cell death not reduced cell migration, suggesting that these four miRNAs have different biological functions mediated by different gene targets. Other investigations have corroborated our findings on miR-203 [36] and miR-205 [37]. The tumor suppressive role of miR-451a has been demonstrated in cancers of lung [32], breast [38] and brain (glioma cells) [31]. In glioma cells, the effects of miR-451 are mediated by LKB1 in the AMPK pathway, where LKB1 is repressed by targeting CAB39 [31]. Similar to glioma cell lines, our melanoma cells did exhibit a glucose-sensitive decreased cell migration; however, this action was not mediated by CAB39, suggesting the context-dependent action of miRNAs in gene regulation.

Finally, higher expression of let-7i, let-7a and let-7f was detected in lightly melanized melanocytes whereas higher expression of miR-100, miR-590, miR-30b was found in heavily melanized melanocytes. These findings implicate miRNAs as important regulators of skin pigmentation in skin under normal physiological condition.

## Materials and Methods

### Study population and clinical samples

The small RNA libraries (18–30 nt) were prepared from 19 mostly formalin-fixed paraffin-embedded (FFPE) specimens consisting of PCM biopsies/excisions, matched NS and CN; and 9 samples from cultured primary melanocytes (CMEL), cultured primary melanoma cell lines (CPM) and cultured metastatic melanoma cell lines (CMM), as described [14]. For qRT-PCR validation, the study cohorts consisted of patients with NS (n = 19), CN (n = 16), melanoma in situ (MIS, n = 17) and PCM (n = 30) undergoing curative treatment at the Dermatology department at University of Connecticut Health Center (UCHC) from 2003 onward (Table S3 in File S1). For miRNA in situ hybridization (MISH), at least 23 additional biopsies of NS (n = 3), CN (n = 10) and PCM (n = 10) were selected. A board certified pathologist/dermatopathologist confirmed all rendered diagnoses. The institutional review board of the University of Connecticut (UCONN) Health Center approved this protocol.

### Histopathological characterization

For all cohorts, we collected detailed clinicopathologic data on melanomas such as histologic subtype, depth of invasion, ulceration, mitotic index, anatomic level of invasion, tumor infiltrating lymphocytes and regression as described previously [39]. Other histopathologic features such as upward scatter of melanocytes, nesting of melanocytes, cell shape and solar elastosis were recorded as described previously [40]. NS was defined as histologically unremarkable skin, 2 cm away from melanoma in excision specimens. We defined cytological atypia in DN by comparing the nuclear size of atypical melanocytes to that of a mid-layer keratinocyte in the same lesions; if smaller recorded as slight; same size to 1.5× larger as moderate and>2× larger as severe.

### Melanin pigmentation

Pigmentation was defined as melanin accumulation within the constituent melanoma cells and was scored on a four-point scale using 20× and 40× objectives. Dermal melanophages were not considered. We classified melanomas based on the percentage of melanin-containing melanoma cells as described previously [34], [40,]. Pigmentation was scored as follows: absent (amelanotic) if only <5%; faint if 6-25%; moderate if 26–50% and abundant if>51% of the tumor cells contained melanin.

### TaqMan miRNA assay

The expression profile of mature miRNA for let-7a, let-7b, let-7c, let-7e, let-7f, let-7g and let-7i, miR-211, miR-27b, miR-26b, miR-126, miR-30d, miR-365, miR-150, miR451a and miR451a.1 was measured in specimens and cell lines using stem-loop primers for reverse transcription followed by qRT-PCR (TaqMan MicroRNA Assays; Applied Biosystems, Foster City, CA) in a 7500 fast Sequence Detection System (Applied Biosystems). Cycle threshold (Ct) values for each miRNA were normalized vs. small RNA RNU6 (ΔCt) and represented as RQ = 2^−ΔCt^. For each sample, 5 ng of total RNA were used for reverse transcription and 1.33 µl of 15-µl reverse transcription product was used for each qRT-PCR. All experiments were carried out in triplicates with appropriate negative control. The average RQ values were calculated for each sample in the diagnostic group, after all triplicate values passed a quality control (not flagged). To determine the fold RQ difference for a specific miRNA among different cell lines or melanocytes, we normalized the average RQ values from a given cell line per CMELM.

### RNA quantification and size

The yield and quality (260/280 O.D. ratios) of RNA were measured by a spectrophotometer (Nanophotometer, Implen, Germany). Small RNA size was measured using Agilent Small RNA Kit with an Agilent 2100 Bioanalyzer (Agilent Technologies, Waldbronn, Germany).

### miRNA in situ hybridization (MISH)

MISH was performed essentially as previously described [41]. Briefly, FFPE tissue sections were treated with 20 µg/ml proteinase K (Ambion, USA) for 10 min at 37°C, and consequently fixed with 4% paraformaldehyde (Thermo Scientific, Rockford, IL, USA) followed by fixation with 0.16 M 1-ethyl-3-(3-dimethylamino propyl) carbodiimide (EDC, Sigma-Aldrich, St Louis, MO, USA) 0.13 M 1-methylimidazole (Sigma-Aldrich, St Louis, MO, USA). Endogenous peroxidases were blocked with 1% hydrogen peroxide (Sigma-Aldrich, St Louis, MO, USA). Slides were pre-hybridized at 50°C with hybridization buffer followed by hybridization with 80 nM double Digoxigenin (DIG) labeled LNA-modified probe corresponding to mature miR-451a.1 (Exiqon, Copenhagen, Denmark), 80 nM single DIG scramble control or 40 nM single DIG U6 for 1 h at 50°C. Slides were washed thrice with 2X SSC, with the first wash being at hybridization temperature and subsequent washes being at room temperature, blocked with 0.5% Roche blocking reagent (Roche Diagnostics, Indianapolis, IN, USA) then incubated with 1∶100 Anti-DIG-HRP antibody (Novus Biologicals, CO, USA) for 1 h at room temperature. Slides are then washed twice with TBS with 0.1% Tween-20 (TBS-T) and once with TBS, then incubated with 1∶50 Tyramide Signal Amplification (TSA) Plus Cyanine 5 reagent (Perkin Elmer, Norwalk, CT, USA) for 30 min, and washed again as above. Nuclei were counterstained with DRAQ5 (Cell Signaling Technology, Danvers, MA, USA) and the slides were mounted with Prolong Gold (Invitrogen, Carlsbad, CA, USA) mounting medium. Keeping the parameters constant for both miR-451a.1 and scramble probes in all biopsies, images were acquired with a Zeiss LSM ConfoCor3 confocal microscope (Carl Zeiss, Jena, Germany).

### Statistical methods

The qRT-PCR data were plotted and analyzed using statistical analysis software SAS version 9.2. To compare miRNA abundance between groups, ANOVA was first performed to compare logarithmically transformed data. When the overall test of no group differences from the ANOVA was statistically significant (α = 0.05), *post hoc* pairwise comparisons with Tukey's adjustment were performed to identify group pairs that differed significantly in miRNA abundance (family level of significance α = 0.05). Alternatively, Kruskal-Wallis test was performed for overall comparisons among clinic groups (α = 0.05). Bonferroni procedure, based on the ranks of the observations, was then used for multiple pairwise comparisons (family level of significance α = 0.10). To evaluate the correlation of miRNA expression to melanoma histopathology, the samples were subgrouped by histopathologic characteristics. Two-sample t-tests and Wilcoxon-Mann-Whitney test were further performed for subgroup differences.

### Cell lines

Dr. Stanley N. Cohen, Stanford school of medicine, CA, kindly provided A2058, A375P, C32 [8], and A375SM (ATCC). We purchased WM983A (Coriell), WM278 (Coriell), WM35 and WM1552C (Wistar institute, Philadelphia, PA).

### Pigmentation in cultured melanocytes

Three types of primary epidermal melanocytes–isolated from 3 individuals with light, medium and dark skin–were purchased from ScienCell (Carlsbad, CA). All cells were cultured as previously described [8]. By the amount of brown pigment produced in the media and light microscopy, the cultured melanocytes appeared distinctly and differentially pigmented. Melanocytes isolated from a light skin individual did not change the color of medium after 5-7 days; whereas those isolated from a medium color skin produced some brown medium and those from a darkly pigmented individual produced dark brown medium.

### miRNA expression vectors and transfection

We purchased expression vectors containing ∼150 nucleotides of the pre-miRNA corresponding to human miR-144, miR-203, miR-205, miR-211, miR-451, and scramble control (miR-SCR) along with an enhanced green fluorescent protein (eGFP) reporter from GeneCopoeia (Rockville, MD, USA). pCDH (empty vector for control) and pCDH containing miR-144/451 cluster were generously provided by Dr. Sean E. Lawler, Dardinger Laboratory for Neuro-oncology and Neurosciences, Department of Neurological Surgery (The Ohio State University Medical Center and James Comprehensive Cancer Center, Columbus, OH 43210, USA). Cells were seeded at 5×10^5^ per well in a 6-well plate approximately 24 h prior to transfection. Transfections were performed using X-tremeGENE HP DNA Transfection Reagent (Roche Applied Science) following the manufacturer's protocol. Briefly, the transfection mixture contained a total of 2-µg plasmid DNA and 6 µl X-tremeGENE HP in 250 µl Opti-MEM (Invitrogen). After 30 min incubation at room temperature the mixtures were added to the designated wells containing 60 to 70% confluent cell in 2 ml DMEM with 10% FBS and no antibiotics. The plate was incubated for 48 h at 37°C and 5% CO2. The GFP-measured transfection efficiency for A375P and A375SM were ∼75% and for WM983A was ∼30%.

### Western blot detection of CAB39

Cultured cells were lysed in NP40 Cell Lysis Buffer (Invitrogen, Carlsbad, CA) and spun at 16,000xg to extract soluble proteins. Total (50 µg) protein was resolved on a 4-20% Tris-Glycine gradient gel (BioRad) and blotted onto nylon membrane. The membrane was blocked with 5% powdered nonfat milk in TBST buffer for 1 hour, then incubated with 1∶1000 dilution of anti-CAB39 (Cell Signalling) or 1∶25,000 dilution of anti-SDHA antibody (Abcam) overnight at 4°C. The membrane was then washed three times with TBST buffer and incubated with HRP conjugated secondary antibody, washed thrice with TBST, developed with the SuperSignal West Femto Blotting Substrate (Pierce, Rockford, IL) and imaged and analyzed on a Kodak Image Station 4000 MM Pro (Carestream Health, Rochester, NY). Relative band intensity for CAB39 was normalized to succinate dehydrogenase (SDHA) as a loading control.

### Modified cell migration assay

A scratch assay was used for the assessment of the migration of transfected cells, as described previously [42] with slight modification. Briefly, the experimental groups of cells and controls were seeded at 1×10^5^ cell density per well in 6-well plates and transfected as described above and allowed to divide in the growth media containing transfection mix until 90% confluent. One day before the assay the cells were serum deprived using serum-free DMEM (containing 4.5 g/l or 0.3 g/l of glucose). The next day the media was removed and using a sterile 200 yellow pipette tip a migration gap was created by introducing a ‘scratch’ to the adherent layer of cultured cells. Two PBS washes were performed to clean any remaining cell debris and fresh medium with 10% serum was added to the wells. Cells were imaged at time zero post-scratch and then plates were then incubated at 37°C and 5% CO_2_. The imaging of the migration was continued at intervals of 6, 12, 24 h. To be consistent, all images were taken at the same marked location. The gaps in scratched monolayer of green fluorescent protein (GFP) expressing were measured in six different fields. Photographs of scratch assay were taken at a magnification of 10× using an Olympus camera mounted on an Olympus CKX41 inverted microscopes. Using the Photoshop Extended Measurement feature, remaining area between two fronts of the migrating cells in taken micrograph was selected with the Lasso tool and used to measure the cells filling in the scratched area. At least two independent experiments were performed for every miRNA tested.

### Cell invasion assay

Invasion assay was performed using Matrigel and BD Falcon Cell Culture inserts following the manufacturer's protocol, as described previously [43] with slight modification. Briefly, transfected cells with pCDH-miR451/144 and pCDH empty vector were prepared as described above. The media of transfected cells were changed to low glucose (0.3 g/l) and kept at normal glucose (4.5 g/l) growth media for each set. Cells were detached with trypsin and 3×10^5^ cells in 2 ml serum-free medium were added to each permeable transwell (8.0 µm polycarbonate membrane, 6.5 mm insert; Corning Incorporated, Corning, NY, USA) coated with Matrigel basement membrane matrix (1∶5 dilution; BD Biosciences, San Jose, CA, USA). In the outer wells 3 ml DMEM with 10% FCS was pipetted and the Transwell insert were incubated for 24h at 37°C and 5% CO_2_.

## Supporting Information

File S1 Contains the following files: **Figure S1.** miRDeep2 output file showing miR-451a isomiRs in NS3 library. This example provided positions and read counts, showing that miRBase (v18) sequence was not the abundant isomiR; in fact, isomiR1 and isomiR2 were the most abundant sequences. Only the isomiRs with the highest read counts are shown. **Figure S2**. miRDeep2 output file showing miR-451a isomiRs in PCM5 library. Similar to NS3, miRBase (v18) sequence was not the most abundant; instead isomiR1 and isomiR2 were the most abundant sequences. **Figure S3.** Robust miR-451a.1 expression was detected in epidermal keratinocytes in normal skin. (a and b) The signal for miR-451a.1 (red) was readily detected in the nuclei and cytoplasm of epidermal keratinocytes of a normal skin specimen. (c) This signal was not detected in the dermal melanoma cells or the overlying keratinocytes. (d and e) Scramble controls showed no signal in any cell type in normal skin; or (f) invasive melanoma. (h) U6 signal was robustly detected in the nuclei of epidermal keratinocytes and dermal nevus cells; (i) but not in the scramble control. The dotted line represents epidermal-dermal junction. Images for miR-451a.1, scramble and U6 probes were acquired under the same constant parameters. The original magnification was 200X for A, D, H and I; 400X for B, C, E and G. **Figure S4**. miR-451a.1 was not detected in additional scramble controls. (a-c) The signal for miR-451a.1 (red) was not detected in nevus scramble controls or (d-f) melanomas scramble controls were negative. **Tables S1-Table S6.**
(DOCX)

## References

[1] Howlader N NA, Krapcho M, Neyman N, Aminou R, Altekruse SF, et al. (eds). (2013) SEER Stat Fact Sheets: Skin (excl. Basal and Squamous). SEER Cancer Statistics Review, 1975–2009 (Vintage 2009 Populations), National Cancer Institute posted to the SEER web site, 2012 ed. Bethesda, MD: http://seer.cancer.gov/csr/1975_2009_pops09/results_single/sect_01_table.01.pdf.

[2] Davies H, Bignell GR, Cox C, Stephens P, Edkins S, et al (2002) Mutations of the BRAF gene in human cancer. Nature 417: 949–954. 12068308 10.1038/nature00766

[3] Curtin JA, Fridlyand J, Kageshita T, Patel HN, Busam KJ, et al (2005) Distinct sets of genetic alterations in melanoma. N Engl J Med 353: 2135–2147. 16291983 10.1056/NEJMoa050092

[4] Dadras SS (2011) Molecular diagnostics in melanoma: current status and perspectives. Archives of pathology & laboratory medicine 135: 860–869. 21732775 10.5858/2009-0623-RAR1.1

[5] Bartel DP (2004) MicroRNAs: genomics, biogenesis, mechanism, and function. Cell 116: 281–297. 14744438 10.1016/s0092-8674(04)00045-5

[6] Godshalk SE, Paranjape T, Nallur S, Speed W, Chan E, et al (2011) A Variant in a MicroRNA complementary site in the 3′ UTR of the KIT oncogene increases risk of acral melanoma. Oncogene 30: 1542–1550. 21119596 10.1038/onc.2010.536 PMC3069149

[7] Chan E, Patel R, Nallur S, Ratner E, Bacchiocchi A, et al. (2011) MicroRNA signatures differentiate melanoma subtypes. Cell Cycle 10. 10.4161/cc.10.11.15777 PMC3233487 21543894

[8] Ma Z, Swede H, Cassarino D, Fleming E, Fire A, et al (2011) Up-regulated Dicer expression in patients with cutaneous melanoma. PLoS One 6: e20494. 21698147 10.1371/journal.pone.0020494 PMC3117784

[9] Segura MF, Hanniford D, Menendez S, Reavie L, Zou X, et al (2009) Aberrant miR-182 expression promotes melanoma metastasis by repressing FOXO3 and microphthalmia-associated transcription factor. Proceedings of the National Academy of Sciences of the United States of America 106: 1814–1819. 19188590 10.1073/pnas.0808263106 PMC2634798

[10] Levy C, Khaled M, Iliopoulos D, Janas MM, Schubert S, et al (2010) Intronic miR-211 assumes the tumor suppressive function of its host gene in melanoma. Molecular cell 40: 841–849. 21109473 10.1016/j.molcel.2010.11.020 PMC3004467

[11] Mazar J, DeYoung K, Khaitan D, Meister E, Almodovar A, et al (2010) The regulation of miRNA-211 expression and its role in melanoma cell invasiveness. PLoS One 5: e13779. 21072171 10.1371/journal.pone.0013779 PMC2967468

[12] Penna E, Orso F, Cimino D, Tenaglia E, Lembo A, et al. (2011) microRNA-214 contributes to melanoma tumour progression through suppression of TFAP2C. The EMBO journal. 10.1038/emboj.2011.102 PMC3098476 21468029

[13] Segura MF, Belitskaya-Levy I, Rose AE, Zakrzewski J, Gaziel A, et al (2010) Melanoma MicroRNA signature predicts post-recurrence survival. Clinical cancer research: an official journal of the American Association for Cancer Research 16: 1577–1586. 20179230 10.1158/1078-0432.CCR-09-2721 PMC4662869

[14] Kozubek J, Ma Z, Fleming E, Duggan T, Wu R, et al (2013) In-Depth Characterization of microRNA Transcriptome in Melanoma. PLoS One 8: e72699. 24023765 10.1371/journal.pone.0072699 PMC3762816

[15] Morin RD, O′Connor MD, Griffith M, Kuchenbauer F, Delaney A, et al (2008) Application of massively parallel sequencing to microRNA profiling and discovery in human embryonic stem cells. Genome research 18: 610–621. 18285502 10.1101/gr.7179508 PMC2279248

[16] Griffiths-Jones S (2004) The microRNA Registry. Nucleic Acids Res 32: D109–111. 14681370 10.1093/nar/gkh023 PMC308757

[17] Fu H, Tie Y, Xu C, Zhang Z, Zhu J, et al (2005) Identification of human fetal liver miRNAs by a novel method. FEBS letters 579: 3849–3854. 15978578 10.1016/j.febslet.2005.05.064

[18] Vaz C, Ahmad HM, Bharti R, Pandey P, Kumar L, et al (2013) Analysis of the microRNA transcriptome and expression of different isomiRs in human peripheral blood mononuclear cells. BMC research notes 6: 390. 24073671 10.1186/1756-0500-6-390 PMC3851811

[19] Ahn HW, Morin RD, Zhao H, Harris RA, Coarfa C, et al (2010) MicroRNA transcriptome in the newborn mouse ovaries determined by massive parallel sequencing. Molecular human reproduction 16: 463–471. 20215419 10.1093/molehr/gaq017 PMC2882868

[20] Marti E, Pantano L, Banez-Coronel M, Llorens F, Minones-Moyano E, et al (2010) A myriad of miRNA variants in control and Huntington's disease brain regions detected by massively parallel sequencing. Nucleic acids research 38: 7219–7235. 20591823 10.1093/nar/gkq575 PMC2978354

[21] Guo L, Zhao Y, Zhang H, Yang S, Chen F (2013) Close association between paralogous multiple isomiRs and paralogous/orthologues miRNA sequences implicates dominant sequence selection across various animal species. Gene 527: 624–629. 23856130 10.1016/j.gene.2013.06.083

[22] Slominski A, Tobin DJ, Shibahara S, Wortsman J (2004) Melanin pigmentation in mammalian skin and its hormonal regulation. Physiological reviews 84: 1155–1228. 15383650 10.1152/physrev.00044.2003

[23] Hsiao JJ, Fisher DE (2014) The roles of microphthalmia-associated transcription factor and pigmentation in melanoma. Archives of biochemistry and biophysics. 10.1016/j.abb.2014.07.019 PMC4336945 25111671

[24] Slominski A, Zmijewski MA, Pawelek J (2012) L-tyrosine and L-dihydroxyphenylalanine as hormone-like regulators of melanocyte functions. Pigment cell & melanoma research 25: 14–27. 21834848 10.1111/j.1755-148X.2011.00898.x PMC3242935

[25] Slominski AT, Carlson JA (2014) Melanoma resistance: a bright future for academicians and a challenge for patient advocates. Mayo Clinic proceedings 89: 429–433. 24684870 10.1016/j.mayocp.2014.02.009 PMC4050658

[26] Hemesath TJ, Price ER, Takemoto C, Badalian T, Fisher DE (1998) MAP kinase links the transcription factor Microphthalmia to c-Kit signalling in melanocytes. Nature 391: 298–301. 9440696 10.1038/34681

[27] Johannessen CM, Johnson LA, Piccioni F, Townes A, Frederick DT, et al (2013) A melanocyte lineage program confers resistance to MAP kinase pathway inhibition. Nature 504: 138–142. 24185007 10.1038/nature12688 PMC4098832

[28] Rasmussen KD, Simmini S, Abreu-Goodger C, Bartonicek N, Di Giacomo M, et al (2010) The miR-144/451 locus is required for erythroid homeostasis. The Journal of experimental medicine 207: 1351–1358. 20513743 10.1084/jem.20100458 PMC2901075

[29] Masaki S, Ohtsuka R, Abe Y, Muta K, Umemura T (2007) Expression patterns of microRNAs 155 and 451 during normal human erythropoiesis. Biochemical and biophysical research communications 364: 509–514. 17964546 10.1016/j.bbrc.2007.10.077

[30] Bandres E, Bitarte N, Arias F, Agorreta J, Fortes P, et al (2009) microRNA-451 regulates macrophage migration inhibitory factor production and proliferation of gastrointestinal cancer cells. Clinical cancer research: an official journal of the American Association for Cancer Research 15: 2281–2290. 19318487 10.1158/1078-0432.CCR-08-1818

[31] Godlewski J, Nowicki MO, Bronisz A, Nuovo G, Palatini J, et al (2010) MicroRNA-451 regulates LKB1/AMPK signaling and allows adaptation to metabolic stress in glioma cells. Molecular cell 37: 620–632. 20227367 10.1016/j.molcel.2010.02.018 PMC3125113

[32] Wang R, Wang ZX, Yang JS, Pan X, De W, et al (2011) MicroRNA-451 functions as a tumor suppressor in human non-small cell lung cancer by targeting ras-related protein 14 (RAB14). Oncogene 30: 2644–2658. 21358675 10.1038/onc.2010.642

[33] Tian Y, Nan Y, Han L, Zhang A, Wang G, et al (2012) MicroRNA miR-451 downregulates the PI3K/AKT pathway through CAB39 in human glioma. International journal of oncology 40: 1105–1112. 22179124 10.3892/ijo.2011.1306 PMC3584578

[34] Brozyna AA, Jozwicki W, Carlson JA, Slominski AT (2013) Melanogenesis affects overall and disease-free survival in patients with stage III and IV melanoma. Human pathology 44: 2071–2074. 23791398 10.1016/j.humpath.2013.02.022 PMC3783651

[35] Dore LC, Amigo JD, Dos Santos CO, Zhang Z, Gai X, et al (2008) A GATA-1-regulated microRNA locus essential for erythropoiesis. Proceedings of the National Academy of Sciences of the United States of America 105: 3333–3338. 18303114 10.1073/pnas.0712312105 PMC2265118

[36] Poell JB, van Haastert RJ, de Gunst T, Schultz IJ, Gommans WM, et al (2012) A functional screen identifies specific microRNAs capable of inhibiting human melanoma cell viability. PLoS One 7: e43569. 22927992 10.1371/journal.pone.0043569 PMC3425484

[37] Xu Y, Brenn T, Brown ER, Doherty V, Melton DW (2012) Differential expression of microRNAs during melanoma progression: miR-200c, miR-205 and miR-211 are downregulated in melanoma and act as tumour suppressors. British journal of cancer 106: 553–561. 22223089 10.1038/bjc.2011.568 PMC3273359

[38] Bergamaschi A, Katzenellenbogen BS (2012) Tamoxifen downregulation of miR-451 increases 14-3-3zeta and promotes breast cancer cell survival and endocrine resistance. Oncogene 31: 39–47. 21666713 10.1038/onc.2011.223 PMC3175015

[39] Doeden K, Ma Z, Narasimhan B, Swetter SM, Detmar M, et al (2009) Lymphatic invasion in cutaneous melanoma is associated with sentinel lymph node metastasis. Journal of cutaneous pathology 36: 772–780. 19032379 10.1111/j.1600-0560.2008.01166.x

[40] Viros A, Fridlyand J, Bauer J, Lasithiotakis K, Garbe C, et al (2008) Improving melanoma classification by integrating genetic and morphologic features. PLoS Med 5: e120. 18532874 10.1371/journal.pmed.0050120 PMC2408611

[41] Hanna JA, Wimberly H, Kumar S, Slack F, Agarwal S, et al (2012) Quantitative analysis of microRNAs in tissue microarrays by in situ hybridization. BioTechniques 52: 235–245. 22482439 10.2144/000113837 PMC3891915

[42] Zhu N, Lalla R, Eves P, Brown TL, King A, et al (2004) Melanoma cell migration is upregulated by tumour necrosis factor-alpha and suppressed by alpha-melanocyte-stimulating hormone. British journal of cancer 90: 1457–1463. 15054471 10.1038/sj.bjc.6601698 PMC2409669

[43] Hall DMS, Brooks SA (2001) In Vitro Invasion Assay Using Matrigel. In: Brooks SA, Schumacher U, editors. Metastasis Research Protocols, Vol 2. Totowa, New Jersey: Humana Press Inc. pp. 61–70.

