# Supplementary material for: A Novel miR-451a isomiR, Associated with Amelanotypic Phenotype, Acts as a Tumor Suppressor in Melanoma by Retarding Cell Migration and Invasion
Source: PLoS One. 2014 Sep 19;9(9):e107502. doi: 10.1371/journal.pone.0107502 (PMC4169564; doi:10.1371/journal.pone.0107502)
Supplement: File S1 — Contains the following files: Figure S1. miRDeep2 output file showing miR-451a isomiRs in NS3 library. This example provided positions and read counts, showing that miRBase (v18) sequence was not the abundant isomiR; in fact, isomiR1 and isomiR2 were the most abundant sequences. Only the isomiRs with the highest read counts are shown. Figure S2. miRDeep2 output file showing miR-451a isomiRs in PCM5 library. Similar to NS3, miRBase (v18) sequence was not the most abundant; instead isomiR1 and isomiR2 were the most abundant sequences. Figure S3. Robust miR-451a.1 expression was detected in epidermal keratinocytes in normal skin. (a and b) The signal for miR-451a.1 (red) was readily detected in the nuclei and cytoplasm of epidermal keratinocytes of a normal skin specimen. (c) This signal was not detected in the dermal melanoma cells or the overlying keratinocytes. (d and e) Scramble controls showed no signal in any cell type in normal skin; or (f) invasive melanoma. (h) U6 signal was robustly detected in the nuclei of epidermal keratinocytes and dermal nevus cells; (i) but not in the scramble control. The dotted line represents epidermal-dermal junction. Images for miR-451a.1, scramble and U6 probes were acquired under the same constant parameters. The original magnification was 200X for A, D, H and I; 400X for B, C, E and G. Figure S4. miR-451a.1 was not detected in additional scramble controls. (a-c) The signal for miR-451a.1 (red) was not detected in nevus scramble controls or (d-f) melanomas scramble controls were negative. Tables S1-Table S6. (DOCX) [file pone.0107502.s001.docx]

**Supplemental Data**

**Supplemental Tables and Figures**

**Supplemental Table 1. Summarized miRNA sequence counts and percent per total.**

| ***Average miRNA sequence counts*** | | | | | | | |
| --- | --- | --- | --- | --- | --- | --- | --- |
| **Sample** | **NS** | **CN** | **PCM** | **CMELL** | **CMELM** | **CMELD** | **Melanoma cell lines^*^** |
| hsa-miR-451a | 668.0 | 52.5 | 80.8 | 0.0 | 0.0 | 0.0 | 0.0 |
| hsa-miR-144-3p | 216.0 | 6.0 | 31.2 | 0.0 | 8.3 | 0.0 | 0.0 |
| hsa-miR-144-5p | 4.0 | 0.0 | 1.5 | 0.0 | 0.0 | 0.0 | 0.0 |
| Total miRNAs | 26391.8 | 111455.0 | 14315.6 | 13617.0 | 18193.0 | 15572.0 | 9962.7 |
|  | | | | | | | |
| ***Average percent/total miRNAs*** | | | | | | | |
| **Sample** | **NS** | **CN** | **PCM** | **CMELL** | **CMELM** | **CMELD** | **Melanoma cell lines^*^** |
| hsa-miR-451a | 2.1 | 0.0 | 0.7 | 0.0 | 0.0 | 0.0 | 0.0 |
| hsa-miR-144-3p | 0.6 | 0.0 | 0.2 | 0.0 | 0.0 | 0.0 | 0.0 |
| hsa-miR-144-5p | 0.0 | 0.0 | 0.0 | 0.0 | 0.0 | 0.0 | 0.0 |

^*^Melanoma cell lines were A2058, A375P, A375SM, C32, WM35 and WM1552C. CMELL, cultured melanocytes of light skin color. CMELM, cultured melanocytes of medium skin color. CMELD, cultured melanocytes of dark skin color.

**Supplemental Table 2. TaqMan qRT-PCR primer assays for miR-451a isomiRs.**

| >hsa-mir-451a MI0001729  (miRBase v18) | CUUGGGAAUGGCAAGGAAACCGUUACCAUUACUGAGUUUAGUAAUGGUAAUGGUUCUCUUGCUAUACCCAGA |
| --- | --- |
| >hsa-mir-451a MI0001729  (miRBase v17) | ………………..……………AAACCGUUACCAUUACUGAGUUU………………… |
| >has-mir-451a  qRT-PCR assay | ……………………………..AAACCGUUACCAUUACUGAGUUU………………… |
| >has-mir-451a.1  qRT-PCR assay | ……………………………..AAACCGUUACCAUUACUGAGU…………… |

The underlined denotes the sequence for miR-451a in miRBase.

**Supplemental** **Table 3. Clinicopathological characteristics of patient specimens.**

| **Diagnostic Group** | **NS** | **CN** | **DN** | **MIS** | **PCM** |
| --- | --- | --- | --- | --- | --- |
| **Number of Samples** | 19 | 16 | 19 | 17 | 30 |
| Male | 9 | 9 | 6 | 7 | 21 |
| Female | 10 | 7 | 13 | 10 | 9 |
| **Anatomic Site** |  |  |  |  |  |
| Head & Neck | 3 | 2 | 1 | 3 | 9 |
| Upper Extremity | 2 | 1 | 0 | 4 | 4 |
| Trunk | 13 | 12 | 18 | 7 | 14 |
| Lower extremity | 1 | 1 | 0 | 3 | 3 |
| **Age at diagnosis (years)** |  |  |  |  |  |
| Mean | 58.6 | 36.5 | 43.9 | 53.2 | 54.9 |
| Min | 40 | 10 | 20 | 26 | 18 |
| Max | 94 | 68 | 77 | 79 | 94 |
| **Histology Subtype** |  |  |  |  |  |
| Superficial spreading |  |  |  | 8 | 22 |
| Lentigo maligna |  |  |  | 9 | 4 |
| Nodular |  |  |  | 0 | 3 |
| **Vertical growth phase** |  |  |  |  |  |
| Absent |  |  |  | 17 | 15 |
| Present |  |  |  | 0 | 14 |
| **Depth of invasion (mm)** |  |  |  |  |  |
| Mean |  |  |  |  | 1.29 |
| Min |  |  |  |  | 0.12 |
| Max |  |  |  |  | 11.5 |
| **Ulceration** |  |  |  |  |  |
| Absent |  |  |  |  | 29 |
| Present |  |  |  |  | 1 |
| **Mitotic index (/mm^2^)** |  |  |  |  |  |
| 0 |  |  |  |  | 17 |
| 1 |  |  |  |  | 4 |
| 2 |  |  |  |  | 5 |
| 4 |  |  |  |  | 2 |
| 6 |  |  |  |  | 1 |
| **Anatomic level of invasion (Clark’s)** |  |  |  |  |  |
| I |  |  |  |  | 1 |
| II |  |  |  |  | 13 |
| III |  |  |  |  | 6 |
| IV |  |  |  |  | 5 |
| V |  |  |  |  | 1 |
| Mean |  |  |  |  | 2.69 |
| **Tumor Inflammation** |  |  |  |  |  |
| Absent |  |  |  |  | 11 |
| Non-brisk |  |  |  |  | 14 |
| Brisk |  |  |  |  | 5 |
| **Regression** |  |  |  |  |  |
| Absent |  |  |  |  | 24 |
| Present |  |  |  |  | 5 |
| **Morphological Features** |  |  |  |  |  |
| *Scatter of IEM* |  |  |  |  |  |
| Absent |  |  |  | 7 | 11 |
| 1 |  |  |  | 3 | 6 |
| 2 |  |  |  | 3 | 3 |
| 3 |  |  |  | 4 | 8 |
| *Nesting of IEM* |  |  |  |  |  |
| Absent |  |  |  | 11 | 4 |
| 1 |  |  |  | 2 | 8 |
| 2 |  |  |  | 2 | 11 |
| 3 |  |  |  | 2 | 6 |
| *Cell Shapes* |  |  |  |  |  |
| Round |  |  |  | 4 | 7 |
| Ovoid |  |  |  | 11 | 13 |
| Elongated |  |  |  | 0 | 5 |
| Spindled |  |  |  | 2 | 4 |
| **Solar Elastosis** |  |  |  |  |  |
| Absent |  |  |  | 1 | 11 |
| Slight |  |  |  | 6 | 6 |
| Moderate |  |  |  | 5 | 4 |
| Severe |  |  |  | 5 | 7 |
| **Cytoplasmic Melanin** |  |  |  |  |  |
| Absent (amelanotic) |  |  |  | 2 | 2 |
| Faint |  |  |  | 6 | 11 |
| Moderate |  |  |  | 9 | 12 |
| Abundant |  |  |  | 2 | 4 |

NS, normal skin; CN, common nevus; MIS, melanoma in situ; PCM, primary cutaneous melanoma (invasive), IEM, intraepidermal melanocytes.

**Supplemental Table 4. Pairwise statistical comparisons of miRNA levels among diagnostic groups by Tukey and non-parametric methods.**

| **miRNA** | **NS** | **CN** | **DN** | **MIS** | **PCM** |
| --- | --- | --- | --- | --- | --- |
| **miR-451a^t, n^** | * |  |  |  |  |
|  | * |  |  |  |  |
|  | * |  |  |  |  |
|  | * |  |  |  |  |
|  | | | | | |
| **miR-451a.1^t, n^** | * |  |  |  |  |
|  | * |  |  |  |  |
|  | * |  |  |  |  |
|  | * |  |  |  |  |

The pairwise comparisons of the two statistically significant disease groups are shown in the same color. ^*^The group with higher miRNA levels. ^t^Tukey and ^n^Non-parametric methods.

**Supplemental Table 5. Correlation between melanoma clinicopathological characteristics and miR-211, miR-451a and miR-451a.1 expression**

|  | **PCM (n=30)** | | **MIS (n=17)** | |
| --- | --- | --- | --- | --- |
|  | **miRNA** | **Parameter with higher value** | **miRNA** | **Parameter with higher value** |
| **Gender** | miR-211^m^ | Male | NSD |  |
|  | miR-451a^m, t^ |  |  |  |
|  | miR-451a.1^m, t^ |  |  |  |
| **Age at diagnosis** | NSD |  | NSD |  |
| **Histological subtype** | miR-451a^m^ | SSM | NSD |  |
| **Cytoplasmic melanin** | miR-451a.1^m, t^ | Absent to faint | miR-451a^m, t^  miR-451a.1^m^ | Absent to moderate |
| **Upward scatter of IEM** | NSD |  | miR-451a^m^ | Absent |
| **Nesting of IEM** | NSD |  | NSD |  |
| **Solar elastosis** | NSD |  | NSD |  |
| **Cell shape** | NSD |  | NSD |  |
| **Vertical growth phase** | NSD |  |  |  |
| **Depth of invasion (mm)** | NSD |  |  |  |
| **Clark’s level** | NSD |  |  |  |
| **Mitotic index (/mm^2^)** | NSD |  |  |  |
| **Tumor inflammation** | miR-211^m, t^  miR-451a^m, t^ | Present, non-brisk |  |  |

PCM, primary cutaneous melanoma (invasive); MIS, melanoma in situ; NSD, no significant difference for any miRNA. ^m^Statistically significant by Wilcoxon-Mann-Whitney test (alpha =0.05); ^t^Statistically significant by t-test (alpha =0.05). IEM, intraepidermal melanocytes; SSM, superficial spreading melanoma.

**Supplemental Table 6. Association between histopathological phenotype and miR-451a isomiRs in melanoma (n=47)**

| **Melanoma phenotype** | **miR-451a** | **miR-451a.1** |
| --- | --- | --- |
| Melanin | Melanotic | Amelanotic |
| Pagetoid scatter of melanocytes | Absent |  |
| Histological subtype | Superficial spreading |  |
| Tumor inflammation | Present |  |

**Supplemental Table 7. List of used abbreviations**

| **Abbreviations** | **Description** |
| --- | --- |
| NS | Normal skin |
| CN | Common (melanocytic) nevus |
| DN | Dysplastic nevus |
| PCM | Primary cutaneous melanoma |
| qRT-PCR | Quantitative real time polymerase chain reaction |
| NGS | Next generation sequencing |
| FFPE | Formalin-fixed paraffin-embedded |
| CMEL | Cultured primary melanocytes |
| CPM | Cultured primary melanoma cell lines |
| CMM | Cultured metastatic melanoma cell lines |
| isomiR | Isomeric microRNAs |
| miRNA | microRNA |
| LKB1 | Liver kinase B1 |
| AMPK | 5' adenosine monophosphate-activated protein |


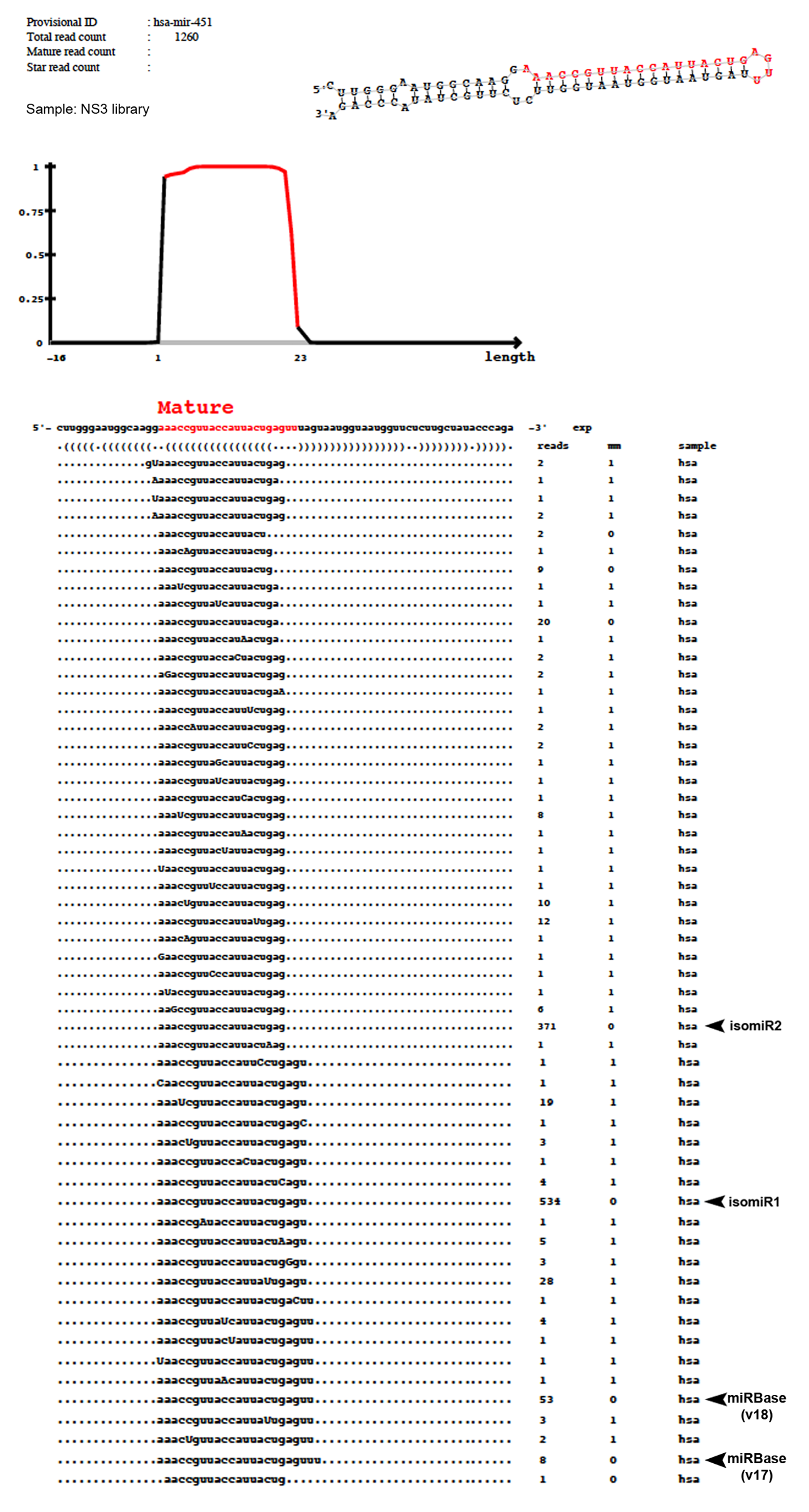


Supplemental Figure 1.


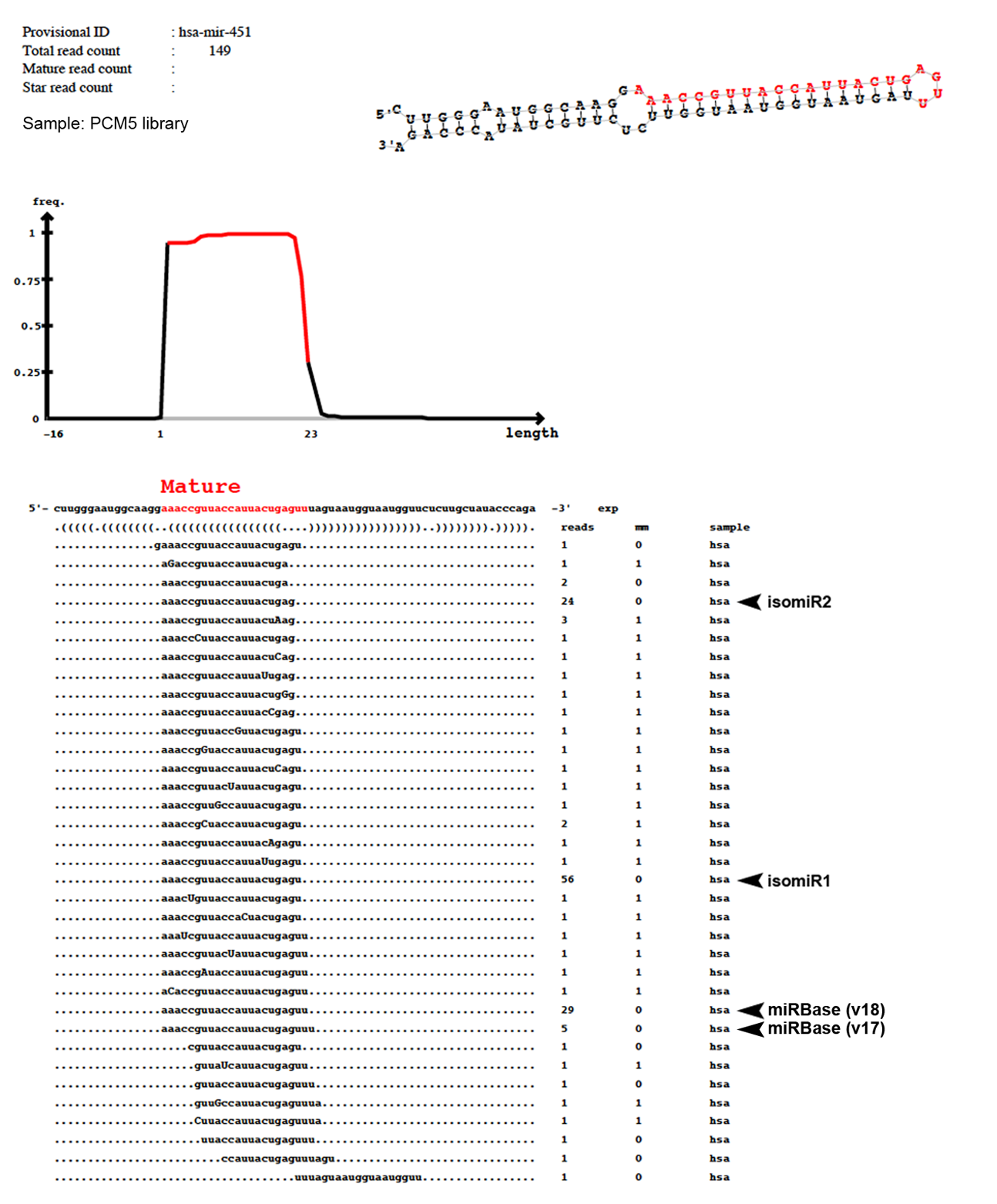


Supplemental Figure 2.


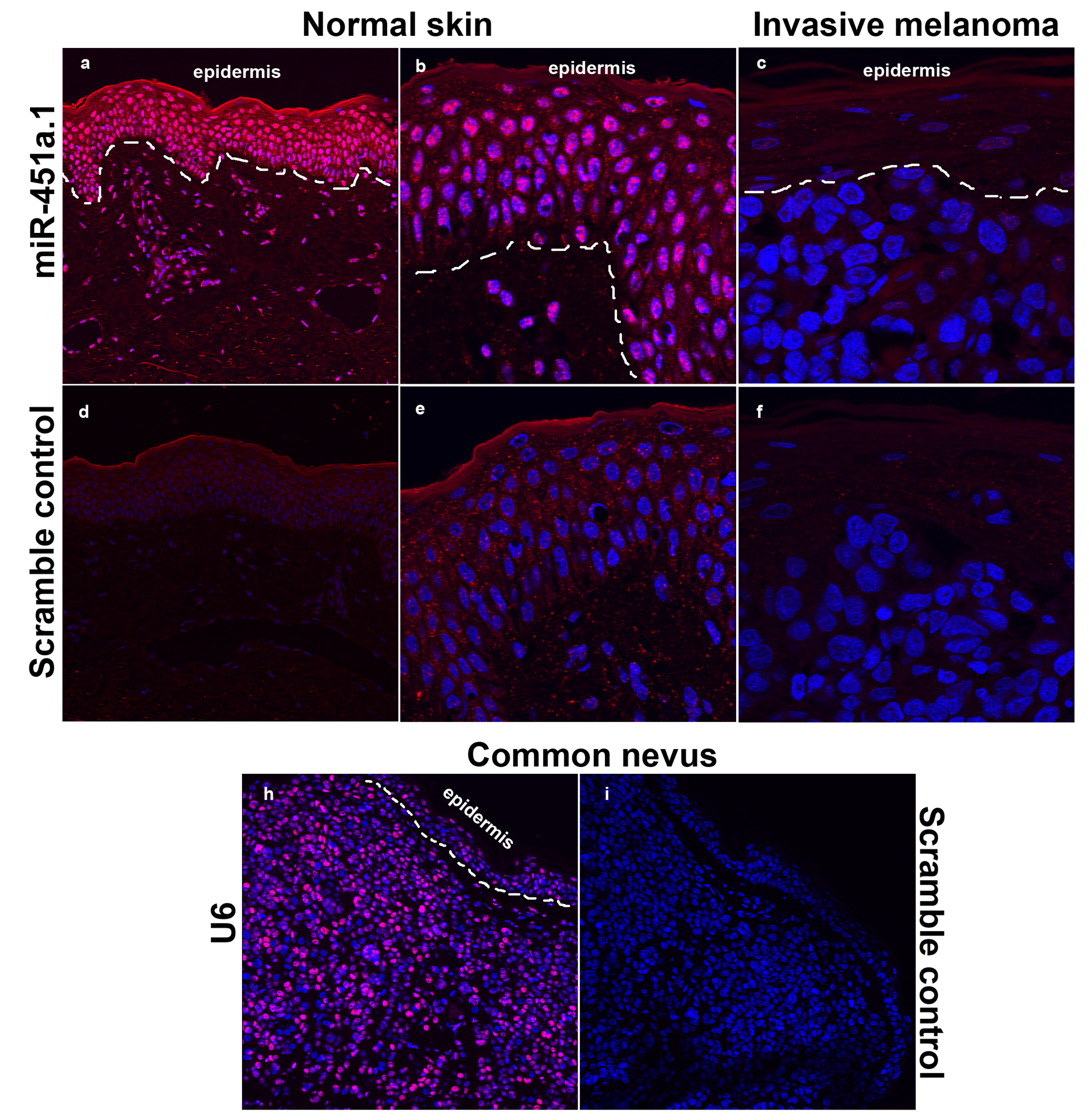


Supplemental Figure 3.


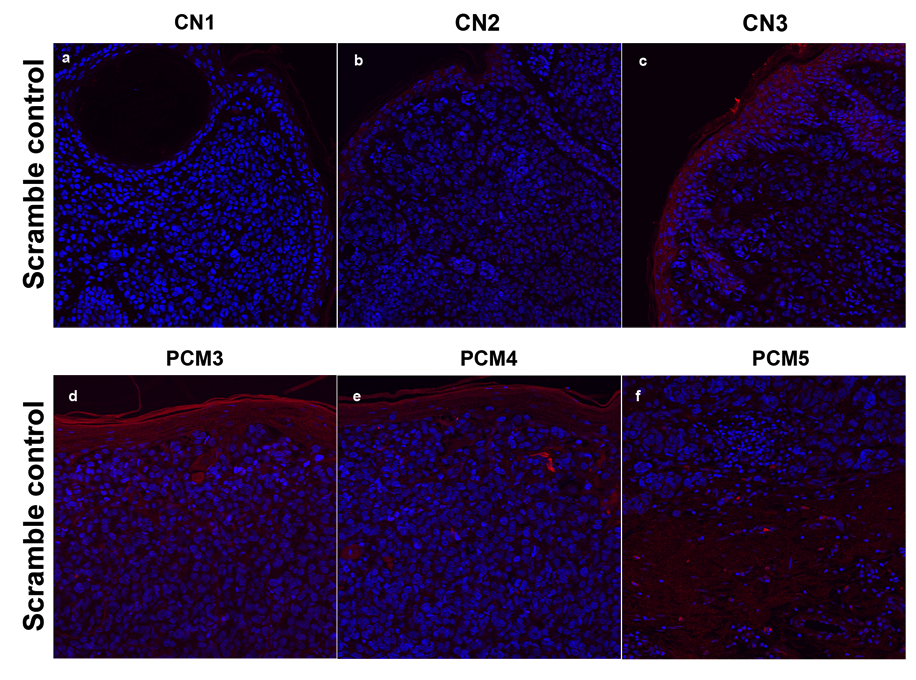


Supplemental Figure 4.
